# Anti-BP180 Autoantibodies Are Present in Stroke and Recognize Human Cutaneous BP180 and BP180-NC16A

**DOI:** 10.3389/fimmu.2019.00236

**Published:** 2019-02-26

**Authors:** Yanan Wang, Xuming Mao, Di Wang, Christoph M. Hammers, Aimee S. Payne, Yiman Wang, Hongzhong Jin, Bin Peng, Li Li

**Affiliations:** ^1^Department of Dermatology, Peking Union Medical College Hospital, Peking Union Medical College, Chinese Academy Medical Science, Beijing, China; ^2^Department of Dermatology, University of Pennsylvania, Philadelphia, PA, United States; ^3^Department of Dermatology, University of Luebeck, Luebeck, Germany; ^4^Department of Neurology, Peking Union Medical College Hospital, Peking Union Medical College, Chinese Academy Medical Science, Beijing, China

**Keywords:** BP180, anti-BP180 autoantibodies, BP180-NC16A, bullous pemphigoid, stroke

## Abstract

**Objective:** Current evidence has revealed a significant association between bullous pemphigoid (BP) and neurological diseases (ND), including stroke, but the incidence of BP autoantibodies in patients with stroke has not previously been investigated. Our study aimed to assess BP antigen-specific antibodies in stroke patients.

**Design:** One hundred patients with stroke and 100 matched healthy controls were randomly selected for measurement of anti-BP180/BP230 IgG autoantibodies by enzyme-linked immunosorbent assay (ELISA), salt-split indirect immunofluorescence (IIF), and immunoblotting against human cutaneous BP180 and BP180-NC16A.

**Results:** Anti-BP180 autoantibodies were found in 14 (14.0%) patients with stroke and 5 (5.0%) of controls by ELISA (*p* < 0.05). Sera from 13 (13.0%) patients with stroke and 3 (3.0%) controls reacted with 180-kDa proteins from human epidermal extract (*p* < 0.05). 11 (11.0%) of stroke and 2 (2.0%) of control sera recognized the human recombinant full length BP180 and NC16A (*p* < 0.05). The anti-BP180-positive patients were significantly younger than the negative patients at the time of stroke (*p* < 0.001).

**Conclusion:** Development of anti-BP180 autoantibodies occurs at a higher frequency after stroke, suggesting BP180 as a relatively common autoantigen after stroke and providing novel insights into BP pathogenesis in aging.

## Introduction

Bullous pemphigoid (BP) is an autoimmune blistering skin disorder most commonly found in the elderly (1). It is characterized by circulating and tissue-bound autoantibodies directed against two hemidesmosomal components: the transmembrane BP180 (collagen XVII, BPAG2) protein, and the plakin family protein BP230 (BPAG1). The skin-specific BP180 molecules locate primarily at the basement membrane zone (BMZ) (2), and the neuronal form of BP180 was observed mainly in cytoplasm, which is highly expressed in the hypoglossal nucleus, oculomotor nucleus, and pyramidal cells of the hippocampal regions in human brain (3). Expression of tissue-specific isoforms of BP180, such as the skin and neuronal BP180, is attributable to alternative splicing and variation in translational start sites of the gene transcripts, although the exact difference between these isoforms was not clearly demonstrated (3, 4). Anti-BP180 IgG autoantibodies play a key role in blister formation and correlate with disease activity in BP, particularly at the time of diagnosis and at disease flare (2, 5). These autoantibodies mainly target immunodominant epitopes of BP180 localized in the extracellular non-collagenous 16A (NC16A) domain (2). The majority of BP patients also react with the intracellular antigen BP230, which is thought to result from a secondary immune reaction after tissue damage (6).

A significant association between BP and neurological diseases (ND) (stroke, dementia, Parkinson disease, epilepsy, and schizophrenia) has been fully supported by a series of previous studies (7–9). Development of BP autoantibodies against both the skin and neuronal forms of antigens in BP with ND might be associated with aging-related dysfunction of the immune system in the elderly population. Alternatively, the autoimmune response might be triggered by chronic inflammatory changes or tissue damage in ND, which exposes antigens from the brain to the immune system.

Stroke is one of the most common forms of ND, which often presents as a life-threatening condition and is the second leading cause of death worldwide. It is characterized by an acute onset and considerably severe tissue damage in the brain. Recently, a study including 12,607 patients with first-ever stroke has revealed that 38 (0.3%) patients developed BP in a median of 3.5 years, while only eight people (0.06%) had BP in a median of 3.7 years in the control group (10). In addition, a population-based case-control study has shown that there was a 2-fold increase in risk of developing BP in those with acute ischemic stroke in the UK (11). In the current study, we aimed to quantitatively determine the level and estimate the positive rate of BP autoantibodies in stroke patients, which may shed light on the mechanism for the high incidence of BP in the ND.

## Methods

### Patient Samples

Following the principles of the Declaration of Helsinki this study was approved by the Ethical Committee of Peking Union Medical College Hospital and informed consent was obtained from all patients and unaffected individuals. Patients with stroke were from the Department of Neurology, Peking Union Medical College Hospital. These included 82 patients with ischemic, 5 with hemorrhagic, and 13 with both ischemic and hemorrhagic stroke, who had been diagnosed with stroke for 1 week to 19 years before our study. The patients were recruited for review and serum samples were collected during July 2014 to October 2016. Stroke was diagnosed based on medical history, clinical symptoms, and results of neuroimaging in the Department of Neurology. The age- and sex- matched control group was comprised of patients attending the hospital for surgery (benign neoplasms resection) in the Department of General Surgery from 2014 to 2016. The average age of stroke and control patients is 66 years for both populations, with a proportion of male patients of 69 and 74%, respectively. Patients with neurological diseases (other cerebrovascular disease, Parkinson's disease, dementia, multiple sclerosis, and amyotrophic lateral sclerosis) and skin diseases (bullous skin disease, dermatitis, eczema, and other autoimmune diseases) were excluded from the control group after reviewing medical records. Normal human foreskin was obtained from patients receiving circumcision in the Department of Urology.

### BP180 and BP230 Enzyme-Linked Immunosorbent Analysis (ELISA)

Anti-BP180/BP230 IgG autoantibodies in the sera samples of patients and healthy controls were detected by commercially available ELISAs for human BP180 (NC16A domain) IgG (MEASACUP BP180, MBL, Japan) and BP230 N-and C-terminal domain IgG (BP230 ELISA kit, MBL, Japan), according to the manufacturer's instructions (based on a cut-off value >9 U/ml).

### Immunoblotting

Protein extract preparations, polyacrylamide gel electrophoresis, and immunoblotting were performed as previously described (12). Briefly, human foreskin samples were treated with 1 M NaCl (24 h) and the epidermis were subjected to Cell Lysis Solution (Thermo Fisher Scientific, Massachusetts, U.S.A). Following homogenization, ice-incubation (30 min) and centrifugation (4,700 rpm, 4°C, 15 min), the supernatant was collected and mixed with loading buffer. Primers were designed according to the full length BP180/NC16A DNA sequence to amplify the target gene fragments by PCR and sequence for FLAG tag was incorporated into the cDNA. Then the human full length BP180/NC16A gene fragments were subcloned into the pcDNA3.1 mammalian expression vector. HEK 293 cells were transiently transfected with the plasmids and lipofectamine (Life Technologies, Carlsbad, CA, USA) as per the manufacturer's instructions, followed by lysis of the cells in a lysis buffer (50 mM Tris (PH8.0), 300 mM NaCl, 1% Triton X-100, 1 mM DTT, 5% glycerol). The proteins in lysates were purified using FLAG peptide affinity chromatography and peptide elution. Expression of the target proteins were confirmed by western-blot (anti-FLAG tag antibody) and quantified by the protein quantification assay kit with final concentrations of 600–1,000 μg/ml. Proteins were subjected to 8% SDS-PAGE gels under denaturing conditions, transferred onto a PVDF membrane (Thermo Fisher Scientific, Massachusetts, U.S.A), and incubated with 0.5 ug/ul human serum samples as the primary antibody and anti-human lgG-HRP (Abcam, Cambridge, Britain) as the secondary antibody. Membranes were developed with detection solution (Merck KGaA, Darmstadt, Germany) and the protein side of the membrane was exposed to an image analysis system (Tanon, Shanghai, China). Anti-human Collagen XVII antibody (Abcam, Cambridge, Britain) (primary antibody) and goat anti-rabbit IgG H&L (HRP) antibodies (Abcam, Cambridge, Britain) (secondary antibody) were used as the positive control to detect BP180-protein in immunoblots.

### Salt Split Indirect Immunofluorescence (IIF)

Five micro molar frozen non-fixed sections of human skin (treated with 1 M NaCl) were blocked (1% BSA in PBS) and human sera in 1:4 to 1:320 dilutions were used as primary antibodies. Rabbit anti-human IgG-FITC (Abcam, Cambridge, Britain) was used as the secondary antibody. The skin sections were then mounted with glycerol/PBS (2:1, pH 9.0) and observed under a fluorescence microscope. A clear linear immunostaining on the BMZ was considered positive, while no fluorescence was considered negative.

### Statistical Analysis

The data involved in the statistical analysis included qualitative analysis, such as BP180 antibody (positive/negative), sex (male/female), complication (yes/no), and frequency of attack (single/ multiple); and quantitative analysis (such as age, BP180/BP230 antibody titer). Specifically, qualitative data were statistically analyzed by chi square test and logistic regression analysis, and the quantitative data were statistically analyzed by *t*-test and rank sum test.

## Results

### BP180 but Not BP230 Autoantibodies Are Significantly Elevated in Patients After Stroke Compared to Unaffected Controls

**Figure 1:**
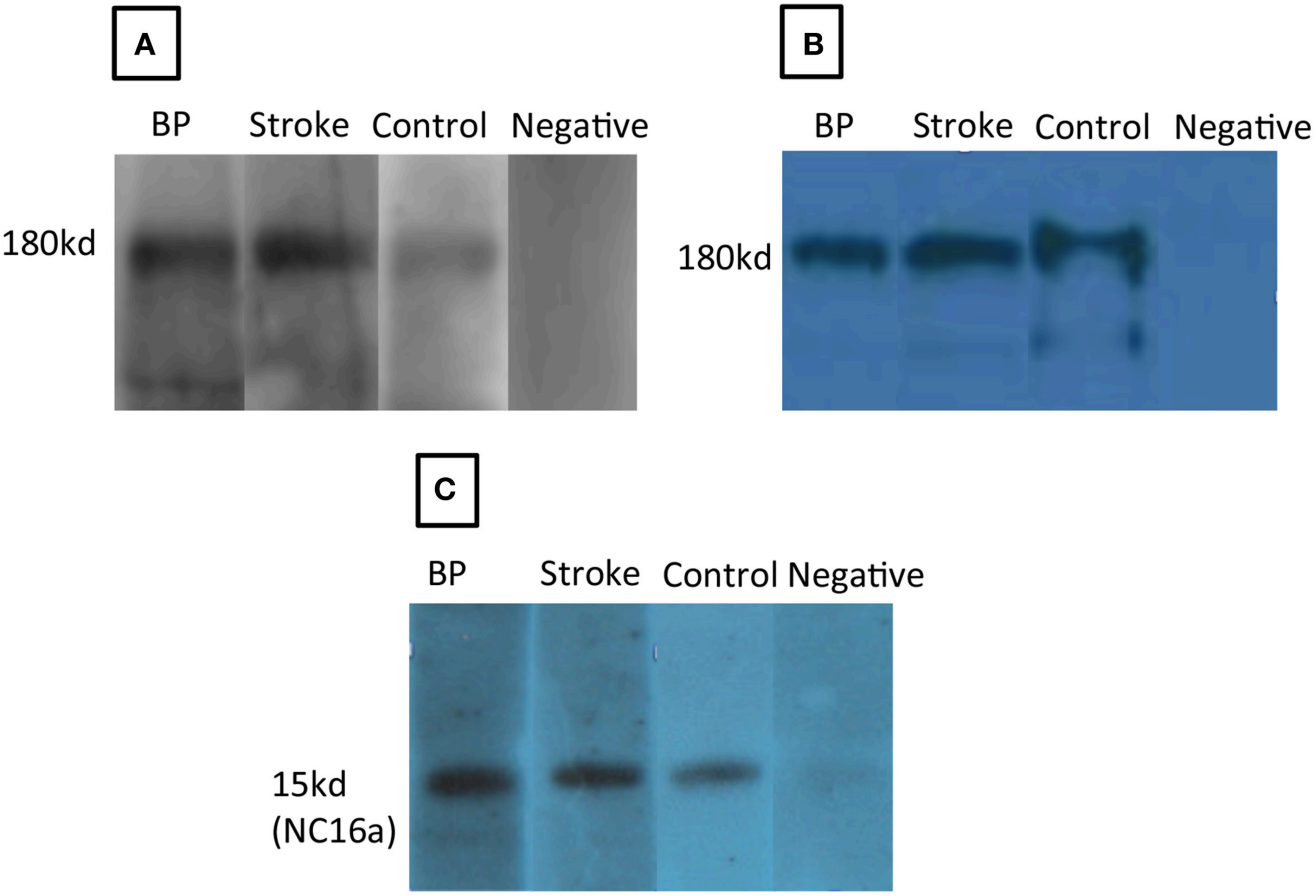
Autoantibodies in the sera of stroke patients react with BP180 and BP180-NC16A in immunoblotting. Serum antibodies from a patient with bullous pemphigoid (BP), a stroke patient (Stroke), or anti-human BP180 antibody (Control) but not a negative control serum (Negative) recognized a 180-kDa protein from human epidermal extract **(A)**, the human recombinant full length BP180 **(B)**, and human recombinant BP180-NC16A **(C)**.

**Figure 2:**
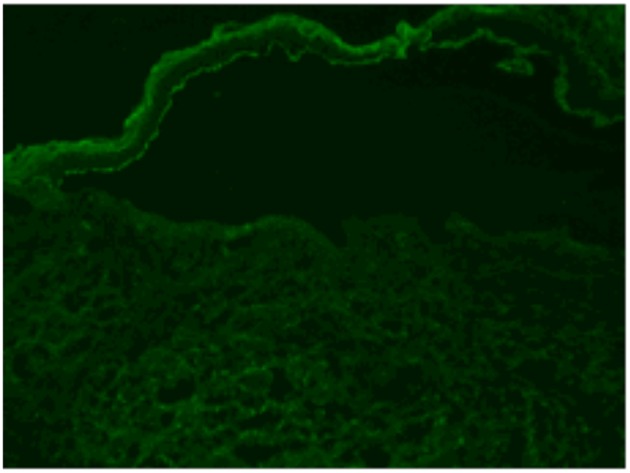
Positive result of salt split IIF. The IgG autoantibodies in the sera from a stroke patients bind to the epidermal side of BMZ (linear deposition of antibodies as shown in green).

Sera from patients with stroke (*n* = 100) (including cerebral infarction and cerebral hemorrhage) and healthy controls (*n* = 100) were collected to examine anti-BP180/BP230 IgG antibodies by ELISA (cut-off value >9 U/ml). The positive rate of anti-BP180 antibody in the stroke cohort (14, 14.0%) was significantly higher than that in controls (5, 5.0%) (*P* = 0.03) (Tables 1, 2). All anti-BP180 IgG positive patients (14 stroke samples and five healthy controls) were further examined by immunoblotting against human epidermal extract, human recombinant full length BP180, and human recombinant NC16A (Tables 1, 2). Sera from 13(13.0%) stroke patients and 3 (3.0%) healthy controls reacted with a 180-kDa protein from the human epidermal extract (*P* = 0.016) (Figure 1A). Sera from 11 (11.0%) patients with stroke and 2(2.0 %) healthy controls recognized both of the human recombinant full length BP180 (*P* = 0.018) (Figure 1B) and human recombinant NC16A (*P* = 0.018) (Figure 1C). Anti-BP180 positive sera were further tested by salt-split IIF, and only one patient with stroke revealed IgG antibody binding on the epidermal side of BMZ (Figure 2).

**Table 1:** Comparison of the BP autoantibody positive rates between stroke and control.

**Item**	**Stroke**	**Control**	***P*-value**
*n*	100	100	
BP180 ELISA	14(14.0%)	5(5.0%)	0.03^*^
**IMMUNOBLOTTING**			
Human epidermal extract	13(13.0%)	3(3.0%)	0.016^*^
Human recombinant full length BP180	11(11.0%)	2(2.0%)	0.018^*^
Human recombinant NC16A	11(11.0%)	2(2.0%)	0.018^*^
Salt split IIF	1(1.0%)	0(0.0%)	0.316

ELISA, enzyme-linked immunosorbent assay; IIF, indirect immunofluorescence. Asterisk

“*” *denotes a statistical significance*.

**Table 2:** Immunological testing results of anti-BP180 positive patients.

**No**.	**Diagnosis**	**BP180 ELISA (U/ml)**	**Immunoblotting**			**Salt-split IF**
			**Human epidermal extract**	**BP180**	**NC16A**	
1	CI and CH	17	+	+	+	–
2	CI	19	+	–	–	–
3	CI	23	+	+	+	–
4	CI	19	+	–	–	–
5	CI	15	+	+	+	–
6	CI	30	+	+	+	–
7	CI	17	+	+	+	IgG+
8	CH	23	+	+	+	–
9	CI	22	+	+	+	–
10	CI	10	+	+	+	–
11	CI	18	+	+	+	–
12	CI	30	+	+	+	–
13	CI	10	–	–	–	–
14	CI	16	+	+	+	–
15	Control	11	+	+	–	–
16	Control	11	+	+	–	–
17	Control	10	–	–	+	–
18	Control	16	–	–	–	–
19	Control	13	+	–	+	–

*CI, Cerebral infarction; CH, Cerebral hemorrhage; IIF, Indirect immunofluorescence; +, Positive; –, Negative. BP180, Human recombinant full length cutaneous BP180; NC16A, Human recombinant cutaneous BP180-NC16A. BP180 ELISA based on a cut-off value >9 U/ml*.

The positive rate of anti-BP230 antibodies had no statistical difference between the stroke (14, 14.0%) and control groups (15, 15.0%) by ELISA.

### The Appearance of Serum BP180 Autoantibodies Is Not Associated With Clinical Onset of BP During Short-Term Follow up

Anti-BP180 autoantibody titers were significantly higher in the stroke group (19.2 ± 6.07 U/ml) compared to those of control group (12.2 ± 2.39 U/ml; *P* = 0.024). The medical records of anti-BP180 positive patients and controls were reviewed and all patients were followed up until October 2017. In the 1-3-year follow-up period, neither stroke patients nor the controls developed BP-like skin lesions. The 1-3-year survival rate of anti BP180 positive patients with stroke and control were both 100%.

### Younger Stroke Patients Are Significantly More Likely to Develop BP180 Serum Autoreactivity Than Older Stroke Patients

According to statistical analysis, the average age of the anti-BP180 positive group (60.1 years) was significantly lower than that of the anti-BP180 negative group (69.0 years; *P* < 0.001). Among them, the proportion of patients younger than 60 years in the anti-BP180 positive group (8/14, 57.1%) was significantly higher than that of the anti-BP180 negative patients (19/86, 24.4%; *P* = 0.006). The duration of follow up after first stroke attack for the BP180 positive group (7.0 ± 2.94 years) was significantly shorter than that of the anti-BP180 negative group (10.4 ± 6.05 years; *P* < 0.001). There was no significant difference in sex, complications, and stroke attack times between the two groups (Table 3).

**Table 3:** Demographic characteristics of anti-BP180 negative or positive patients in the stroke group.

**Variable**	**BP180 (+)**	**BP180 (–)**	***P*-value**
*n*	14	86	
Sex, M/F	11/3 (79/21%)	58/28(67/33%)	0.540
Age, mean ± sd	60.1 ± 11.20	69.0 ± 11.67	<0.001^*^
<60 years, *n* (%)	8 (57.1)	19 (24.4)	0.006^*^
60–70 years, *n* (%)	4 (28.6)	36 (46.2)	0.395
≥75 years, *n* (%)	2 (14.3)	23 (29.5)	0.508
Stroke attack times ≥2, *n* (%)	6 (42.9)	21 (24.4)	0.194
Duration after first attack ≥1 years, *n* (%)	9 (64.3)	49 (57.0)	0.607
Duration after first attack (y), median	7.0 ± 2.94	10.4 ± 6.05	<0.001^*^

*The numbers and percentages of anti-BP180 negative (–) or positive (+) were listed in the table, respectively. The P-values were calculated by comparing these numbers between these two groups*.

Asterisk

“*” *denotes a statistical significance/*.

*BP180, Human recombinant full length cutaneous BP180*.

## Discussion

Previously we have shown that a significant proportion of serum samples obtained from patients with BP and ND could react with both the human skin and neuronal forms of BP antigens, supporting the existence of BP antigens in the brain of ND patients (12, 13). In the present study, we evaluate the BP autoAb levels in 100 Chinese patients with stroke and 100 unaffected controls, demonstrating that BP autoantibodies are detectable at a higher frequency in stroke patients relative to unaffected controls (14 vs. 5%) and they react with human cutaneous BP180 and BP180-NC16A. The 5% incidence of BP autoantibodies in our control group is consistent with a previous study in 337 healthy Americans (11/297, 3.7%) by ELISA (14). These observations raise the possibility that BP 180 acts as a shared autoantigen in both stroke and BP. We speculate that severe damage or alterations in the human central nervous system (CNS) during the course of stroke could release or expose the neuronal isoform of BP180, thus triggering an immune reaction that may eventually lead to BP and cutaneous damage (12, 13).

In our study, only one stroke patient positive both for BP autoantibodies by ELISA and immunoblotting (human BP180 and BP180-NC16A) showed binding of IgG autoantibodies to the epidermal side of the BMZ, consistent with works by Messingham et al. (15), Tuusa et al. (16), and Kokkonen et al. (17) in Alzheimer's disease, multiple sclerosis, and Parkinson's disease, respectively. Our results suggest that that these autoantibodies were not able to bind the conformational epitope of skin BP180 but the linear protein epitope due to release of the neuronal form, indicating that epitopes bound by BP patient antibodies and stroke patient antibodies do differ. The stroke patient antibodies may bind to epitopes only present in the denatured skin BP180, except for the one case.

Notably, we found that anti-BP180 positive stroke patients (60.1years) were significantly younger than anti-BP180 negative stroke patients (69.0 years; *P* < 0.001), suggesting that young age might be a risk factor for stroke patients to develop BP. The proportion of patients younger than 60 years in the anti-BP180 positive patients (8/14, 57.1%) was significantly higher than that in the anti-BP180 negative patients (19/86, 24.4%; *P* = 0.006) (Table 3). The duration of follow-up after first stroke attack of anti-BP180 positive patients (7.0 ± 2.94 years) was significantly shorter than that of anti-BP180 negative patients (10.4 ± 6.05 years; *P* < 0.001), further supporting that younger stroke patients with shorter duration after first attack are more likely to develop BP antibodies. This may be due to strong immune responses in the early stage after stroke, whereas there is down-regulation of the autoimmune response in the late or recovery stage of stroke (18).

During our follow-up, neither the anti-BP180/BP230 positive stroke patients nor the controls exhibited BP-like skin lesions, in accordance with a previous study that showed none of the anti-BP180/BP230 antibody positive individuals had BP-like skin lesions (14). More recently, Kokkonen et al. showed BP180 autoantibodies were found in 18% of patients with Alzheimer's disease and 3% of controls (*P* = 0.019), while none of them had BP-like lesions (17). The reason why anti-BP antibody positive patients had no BP-like lesions may be due to a few possibilities. First, titers of anti-BP antibodies in these subjects may be too low to cause cutaneous lesions. Second, some patients may be misdiagnosed because of atypical lesions. In about 20% of BP patients, atypical lesions arise as prurigo-like nodules, intertrigo-like pemphigoid, and localized forms. In about 10–20% of BP patients, disease onset is preceded by a prodromal phase of weeks to months with pruritus, excoriations, and eczematous lesions, and some patients never develop blisters (19). Third, the time of follow-up is not long enough and the study sample is not large enough to develop BP. Future studies with longer clinical follow-up and larger samples will be necessary to clarify the association of BP autoantibody development with overt clinical disease.

The positive rate of anti-BP180 ELISA in stroke and control groups was a little higher than that of immunoblotting in our study (Table 1). A meta-analysis described ELISA as a quantitative test with high sensitivity and specificity (87 and 98–100%, respectively) for diagnosis of BP (20), while immunoblotting was a semi-quantitative test. Therefore, the ELISA positive stroke patient with low titers may have a negative test by immunoblotting. It is also important to note that for BP180, 7.8% of BP sera react exclusively to regions of BP180 outside of the NC16A region, which would not be identified by using the commercially available BP180 NC16A ELISA test. Thus, a negative test should be closely followed up with DIF and/or IIF (21). There was no ELISA negative patients that were positive for IIF in our study.

Anti-BP180 autoantibody values were significantly higher in the stroke group (19.2 ± 6.07 U/ml) compared to those in the control group by ELISA (12.2 ± 2.39 U/ml; *P* = 0.024). As the anti-BP180 antibodies are correlated with the disease activity of BP, we considered that the difference between the two groups is of clinical significance and may herald the development of BP in the future. Jedlickova et al. found that male stroke patients were more likely to have BP (22). In our study the proportion of male anti-BP180 positive patients (11/14, 84.6%) showed no significant difference compared to controls (4/5, 80.0%; *P* > 0.999).

We conclude that anti-BP180 autoantibodies could be detected at a higher rate in stroke patients than age- and sex- matched controls, supporting that BP180 could serve as a shared autoantigen in both stroke and BP. Our data suggest young age in stroke could be a risk factor for later developing BP and provide a molecular mechanism for BP associated with ND (23). The present study is limited by a relatively short follow-up time so that, although long enough to detect significant differences in serum reactivity, may not have been long enough to allow for the subsequent development of BP skin lesions. Because of the low incidence of BP, a larger sample size is also necessary to detect the clinical onset of BP after stroke. In the future, cell and animal models may be used to help further clarify why BP is significantly associated with aging and neurologic diseases.

## Author Contributions

YaW performed most of the experiments, analyzed the data, and wrote the manuscript. LL, BP, and HJ conceived the project and supervised the study. LL, YaW, and DW designed the experiments. XM interpreted data and revised the manuscript. LL, BP, CH, AP, and YiW discussed the results and revised the manuscript.

### Conflict of Interest Statement

The authors declare that the research was conducted in the absence of any commercial or financial relationships that could be construed as a potential conflict of interest.
